# STAT3 is activated in multicellular spheroids of colon carcinoma cells and mediates expression of IRF9 and interferon stimulated genes

**DOI:** 10.1038/s41598-018-37294-z

**Published:** 2019-01-24

**Authors:** Elin Edsbäcker, Jason T. Serviss, Iryna Kolosenko, Caroline Palm-Apergi, Angelo De Milito, Katja Pokrovskaja Tamm

**Affiliations:** 1 Department of Oncology-Pathology, Cancer Center Karolinska, Karolinska Institutet, Stockholm, Sweden; 2 Department of Laboratory Medicine, Clinical Research Center, Karolinska Institutet, Stockholm, Sweden

## Abstract

Three-dimensional cell cultures, such as multicellular spheroids (MCS), reflect the *in vivo* architecture of solid tumours and multicellular drug resistance. We previously identified interferon regulatory factor 9 (IRF9) to be responsible for the up-regulation of a subset of interferon (IFN)-stimulated genes (ISGs) in MCS of colon carcinoma cells. This set of ISGs closely resembled a previously identified IFN-related DNA-damage resistance signature (IRDS) that was correlated to resistance to chemo- and radiotherapy. In this study we found that transcription factor STAT3 is activated upstream of IRF9 and binds to the IRF9 promoter in MCS of HCT116 colorectal carcinoma cells. Transferring conditioned media (CM) from high cell density conditions to non-confluent cells resulted in STAT3 activation and increased expression of IRF9 and a panel of IRDS genes, also observed in MCS, suggesting the involvement of a soluble factor. Furthermore, we identified gp130/JAK signalling to be responsible for STAT3 activation, IRF9, and IRDS gene expression in MCS and by CM. Our data suggests a novel mechanism where STAT3 is activated in high cell density conditions resulting in increased expression of IRF9 and, in turn, IRDS genes, underlining a mechanism by which drug resistance is regulated.

## Introduction

Interferon (IFN) signalling plays a critical role in the immune response and regulates pathways involved in antiviral defence, proliferation and apoptosis. Several publications have demonstrated that downregulation of different components of the IFN signalling pathway correlates to tumour development and metastasis, establishing a tumour-suppressive role of IFNs^1–3^. On the other hand, high expression of a subset of IFN-stimulated genes (ISGs), referred to as the IFN-related DNA-damage resistance signature (IRDS), is correlated to therapy resistance, poor overall prognosis, and has been identified in samples from patients with glioma, head and neck, prostate, lung, and breast cancer^4,5^. These contradictive effects suggest a multifaceted involvement of ISGs in cancer and that the activity of the IFN signalling pathway and its effect on tumour progression may vary between types of cancer and possibly also with the stage and/or grade.

The IRDS was first identified in 2004 by Khodarev *et al*. in a human tumour xenograft model where the tumours were made radio-resistant by subjection to repeated cycles of radiation. Signal transducer and activator of transcription (STAT) 1 and the IRDS subset of ISGs were found to be upregulated in the radio-resistant tumours compared to the radiosensitive^5^. This set of genes was later found to be induced in chemo- and radio-resistant cancer cell lines of different origin^6–9^.

The mechanism of IRDS induction in resistant tumours is not fully understood. The above-mentioned studies propose that STAT1 is the main driver of IRDS expression and resistance. IFN regulatory factor 9 (IRF9), phosphorylated STAT1, and phosphorylated STAT2 together form a transcription factor complex termed interferon-stimulated gene factor 3 (ISGF3) that binds to the interferon-stimulated response element (ISRE) and induces expression of ISGs in response to type I IFNs. In a study by Luker *et al*., STAT1, STAT2, and IRF9 were found to be upregulated in paclitaxel resistant MCF7 breast cancer cells in the absence of IFNs^10^. However, transient transfection of IRF9 alone, but not of STAT1 or STAT2, significantly reduced the sensitivity to paclitaxel suggesting that IRF9 is the main driver of resistance in this system. The authors also observed IRF9 overexpression in donor-matched pre-treatment patient samples of breast and uterine tumours compared to the normal tissue^10^. Furthermore, a fibrosarcoma cell line lacking functional IRF9 exhibited enhanced sensitivity to several chemotherapeutic drugs^11^. Collectively, these results indicate that increased IRF9 expression is one of the mechanisms underlying resistance to therapy.

Cancer cells cultured as multicellular spheroids reflect the *in vivo* architecture of solid tumours and are less sensitive to chemotherapeutic drugs, a phenomenon known as multicellular resistance^12^. There are multiple mechanisms that contribute to multicellular resistance; some can be attributed to the structure of the spheroid, others can also be observed in confluent monolayer culture and are referred to as mechanisms of contact resistance^13^. We previously showed that STAT1, STAT2 and IRF9, along with a majority of the IRDS genes, were induced in HCT116 colorectal carcinoma cells grown as MCS^14^. IRF9-mediated upregulation of a representative panel of IRDS genes was also identified in monolayer cultures when grown to confluency^14^. In line with Luker *et al*., we found that overexpression of IRF9 alone triggered the expression of the panel of IRDS genes in the absence of IFNs and conferred resistance to chemotherapeutic drugs^10,14^.

IRF9 transcription is known to be induced by cytokines, for example IFNs and Interleukin 6 (IL-6), but the mechanisms behind IRF9 induction in MCS or other high cell density conditions have not been investigated^15–17^. High cell density has also been demonstrated to result in maintained STAT3 activity in melanoma, breast, bladder, and head and neck squamous cell carcinoma cell lines, although a link between STAT3 activity and IRF9 transcription has not been identified^18,19^. STAT3 activation occurs downstream of receptor- and non-receptor tyrosine kinases, for example Janus activated kinases (JAKs), which phosphorylate STAT3 on tyrosine 705 (p-STAT3). This triggers homo-dimerization and translocation to the nucleus where STAT3 homodimer binds to its response element at the promoter of genes, thus regulating their transcription^20^. IL-6 is a well-known activator of STAT3 and has been linked to the pathogenesis of several types of cancer, including colorectal cancer^21^. This cytokine can be produced by the tumour cells or various cells in the tumour microenvironment and it binds to either the membrane bound IL-6 receptor alpha or a soluble form of the receptor, which then forms a complex with receptor subunit glycoprotein 130 (gp130), thus enabling downstream signalling^22^. IL-6 belongs to a family consisting of 8 cytokines that all signal through gp130 and can activate STAT3^23^. STAT3 activation results in several pro-tumorigenic outcomes such as proliferation, inhibition of apoptosis, epithelial-mesenchymal transition (EMT), tumour angiogenesis, and tumour-associated inflammation^24^. Although we did not investigate the activity of STAT3 in our previous study, increased levels of STAT3 mRNA in MCS of HCT116 cells were observed^14^. Herein we present evidence of a novel mechanism where STAT3 activation in high cell density conditions leads to increased expression of IRF9 and subsequent IRDS gene expression, shedding light on one mechanism by which drug resistance is regulated.

## Results

### STAT3 phosphorylation and IRF9/IRDS expression are induced in MCS

**Figure 1:**
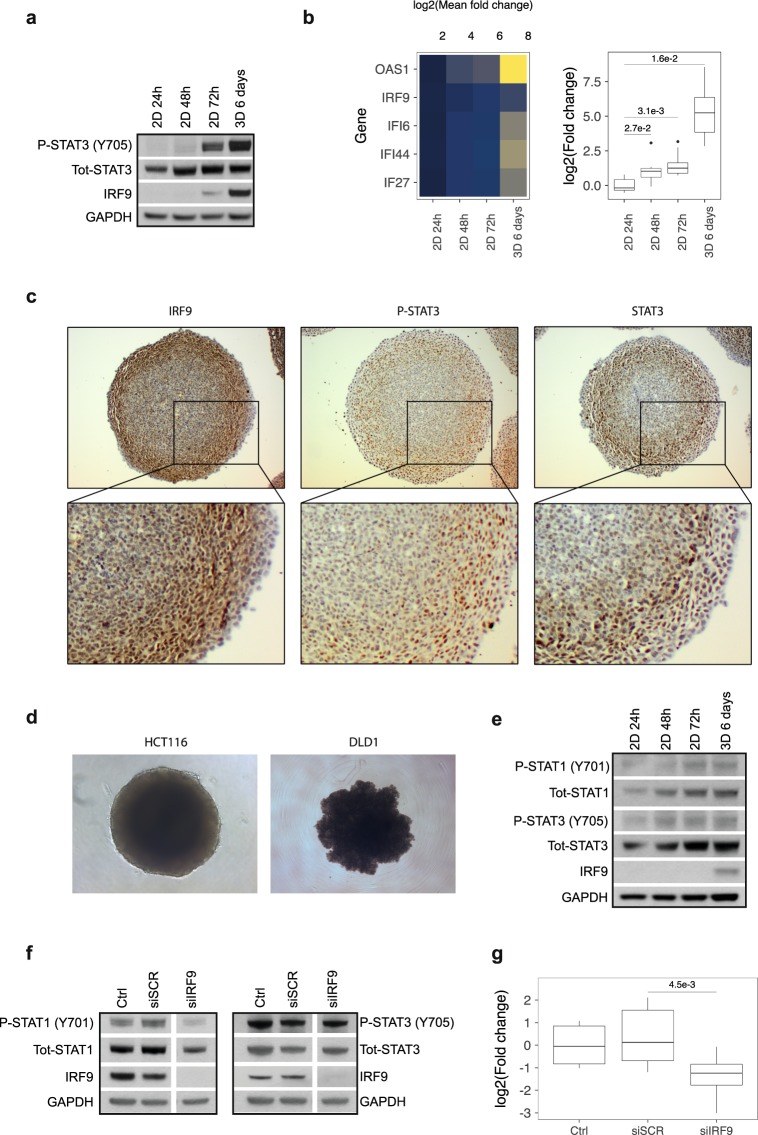
Activation of STAT3 and induction of IRF9 and IRDS genes in MCS. (**a**,**b**) HCT116 cells cultured as MCS (3D) for 6 days compared to cells in monolayer (2D) harvested at indicated time points was subjected to (**a**) Western blot analysis of tyrosine-phosphorylation and protein levels of STAT3 and IRF9 and (**b**) qRT-PCR analysis of mRNA levels of the indicated IRDS genes (n = 3). The heatmap is showing the mean mRNA expression of the individual genes (left) and the box plot display mRNA levels of the IRDS panel as a group (right). (**c**) Representative immunohistochemical staining of IRF9, p-STAT3 and total STAT3 in paraffin embedded HCT116 cells cultured as MCS for 6 days. (**d**) Representative images of HCT116 and DLD1 cells grown as MCS for 6 days. (**e**) DLD1 cells cultured as MCS or in 2D and harvested at indicated time points. Protein levels and phosphorylation of STAT1, STAT3 and IRF9 were analysed by Western blot. (**f**,**g**) Cells were transfected with siRNA targeting IRF9 and cultured as MCS for 48 h. (**f**) Western blot analysis showing knockdown of IRF9 and protein and phosphorylation levels of STAT1 and STAT3. The blots in this figure have been cropped; full-length blots are shown in Supplementary Fig. S1e. (**g**) mRNA expression of the panel of IRDS genes were analysed using qRT-PCR (n = 3). Quantification data of Western blots are available in Supplementary Table 2.

Several studies have shown that high cell density conditions lead to activation of STAT3 in a number of cancer cell lines^18,19^. Microarray data obtained in our previous study showed that STAT3 mRNA was induced in MCSs of HCT116 cells^14^. We therefore set out to investigate whether STAT3 is activated in HCT116 MCS and if it can be responsible for the induction of IRF9 and IRDS genes. In non-confluent cells, cultured for 24 h in 2D, very low STAT phosphorylation and no IRF9 protein expression could be detected by Western blot (Fig. 1a and Supplementary Fig. S1a). Hence, we used this condition as the baseline for STAT activation and expression of IRF9 and the IRDS genes. We found that culturing HCT116 cells as MCS led to tyrosine phosphorylation of STAT3 and increased STAT3 total protein levels compared to non-confluent cells (Fig. 1a). STAT3 DNA binding activity was also induced under these conditions (Supplementary Fig. S1b). Additionally, p-STAT3 was induced in confluent 2D cultures (72 h) compared to non-confluent cultures (24–48 h) (Fig. 1a). We have not used time points beyond 72 h (100% confluent) for 2D culture, due to reduced viability of the cells past this point. Tyrosine phosphorylation of both STAT1 and STAT2 was clearly increased in 3D compared to the non-confluent 2D culture (Supplementary Fig. S1a). In accordance with our previous study, we observed increased mRNA levels of IRF9 over time in 2D and, as well, in 3D culture (Fig. 1b, left). IRF9 protein was only detected in 3D and confluent 2D cultures at the 72 h time point (Fig. 1a). Four ISGs (OAS1, IFI6, IFI27, IFI44), which were among the top upregulated genes in MCS in our previous study^14^, were chosen to represent the IRDS signature^4^. We observed an increase in mRNA levels of our panel of IRDS genes both individually (Fig. 1b, left panel) and as a group (Fig. 1b, right panel) over time in 2D culture, and in 3D compared to non-confluent cells.

Immunohistochemistry of MCS cultured for 6 days revealed that IRF9, p-STAT3 and total STAT3 protein all exhibited a similar expression pattern. Negative or cytoplasmic staining was observed in the centre of the sphere while the outer layer displayed positive nuclear staining of either of the proteins (Fig. 1c).

We also investigated the activation and expression of STAT3 and IRF9 in 2D compared to 3D cultures in the colorectal cancer cell line DLD1. While HCT116 cells make compact round spheres using the “hanging drop” technique, DLD1 cells grew in an aggregate with no defined border (Fig. 1d). STAT3 and STAT1 phosphorylation increased in DLD1 cells in 2D and 3D cultures compared to the non-confluent cells at the 24 h time point (Fig. 1e). IRF9 protein was detected only in DLD1 MCS and not in monolayer cells cultured to confluence (72 hours) (Fig. 1e). The mRNA levels of the panel of IRDS genes were not significantly induced (p = 0.05961) as a group in 3D compared to non-confluent cells (data not shown); however, the increase in OAS1, IFI27 and IFI44 individually were all statistically significant (Supplementary Fig. S1c).

Our previous results indicated that IRF9 is required for the induction of STAT1 and the IRDS genes in confluent monolayer culture^14^. Hence, we decided to investigate the effects of IRF9 knockdown on activity and expression of STAT1 and STAT3, as well as the IRDS genes, in HCT116 MCS. We observed no drastic changes in MCS morphology upon efficient IRF9 knockdown (Supplementary Fig. S1d), but the levels of total and p-STAT1 protein were visibly reduced compared to scrambled siRNA control (Fig. 1f, left panel). In contrast, STAT3 expression and phosphorylation were not affected by IRF9 knockdown (Fig. 1f, right panel). IRF9 knockdown in MCS reduced the mRNA expression of our panel of IRDS genes as a group (p = 0.0045) (Fig. 1g) and individually (Supplementary Fig. S1f), confirming previous observations in confluent monolayer culture^14^. These data further strengthen the role of IRF9 in the induction of IRDS genes in conditions of high cell density and show that STAT3 is activated in HCT116 multicellular spheroids up-stream of IRF9 induction.

### Activation of STAT3 and induction of IRF9 in MCS are mediated by gp130/JAK signalling

**Figure 2:**
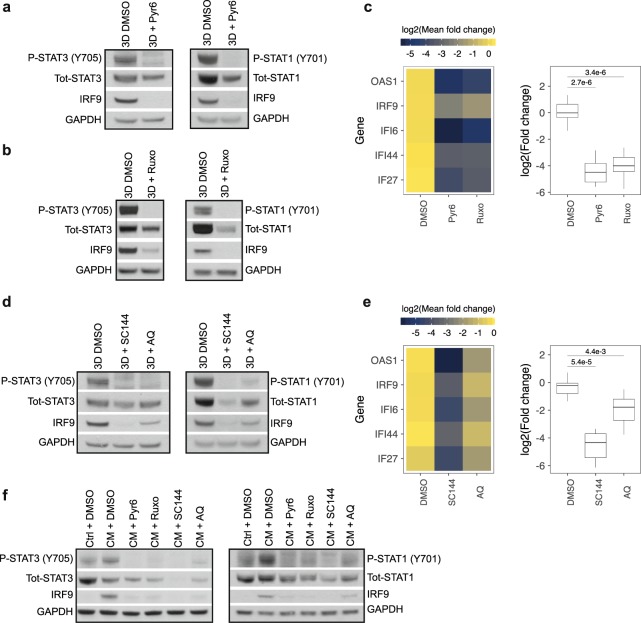
STAT1 and STAT3 activation and IRF9/IRDS genes induction is dependent on gp130/JAK signalling. (**a–c**) HCT116 cells were cultured as MCSs for 6 days in the presence of either DMSO or JAK inhibitors Pyridone 6 (Pyr6) 5 µM or Ruxolitinib (Ruxo) 1 µM. Protein phosphorylation and total levels of STAT1, STAT3 and IRF9 in samples treated with (**a**) Pyr6 or (**b**) Ruxo were analysed by Western blot. (**c**) mRNA expression of the IRDS panel was evaluated by qRT-PCR (n = 3). Mean expression of the individual genes is displayed in the heatmap (left) and mRNA levels of the IRDS panel as a group is shown in the box plot (right). (**d**,**e**) HCT116 cells were cultured as MCSs for 6 days in the presence of DMSO or gp130 inhibitors SC144 (1 µM) or Atovaquone (AQ) (10 µM). (**d**) Protein phosphorylation and total levels of STAT1, STAT3 and IRF9 were analysed by Western blot and (**e**) mRNA expression of the indicated IRDS genes was evaluated by qRT-PCR (n = 2). Heatmap showing the mean mRNA expression of individual genes (left), and the box plot show expression of the IRDS panel as a group (right). (**f**) HCT116 cells seeded at low density were treated with CM and either JAK or gp130 inhibitors for 24 h. Protein phosphorylation and total leves of STAT1, STAT3 and IRF9 in cells treated with JAK inhibitors or gp130 inhibitors were analysed by Western blot. Quantification data of Western blots are available in Supplementary Table 2.

It has been demonstrated that the high cell density-induced activation of STAT3 in melanoma cells is mediated by JAKs^19^. Hence, we sought out to test if JAKs were involved in the activation of STAT3 in MCS of colon cancer cells and whether JAK-inhibition can affect induction of IRF9 and the panel of IRDS genes. To achieve this, HCT116 cells were seeded as MCS and cultured for 6 days in the presence of JAK inhibitors Pyridone 6 or Ruxolitinib. Indeed, STAT3 phosphorylation was abolished and total STAT3 protein levels were decreased by the JAK inhibitors compared to the DMSO control (Fig. 2a,b). Additionally, the expression of IRF9 and total- and phosphorylated-STAT1 were substantially reduced (Fig. 2a,b) and mRNA levels of the IRDS genes were decreased, both individually and as a group (Fig. 2c).

JAKs can be activated by the cytokines that engage receptor-subunit gp130 initiating down-stream signalling^25^. We therefore cultured HCT116 cells as MCS for 6 days in the presence of two different gp130 inhibitors; SC144 or Atovaquone (AQ). SC144 is a small- molecule that induces phosphorylation and deglycosylation of gp130, thus inhibiting downstream signalling^26^. AQ is an FDA-approved antimicrobial agent used to treat pneumocystis pneumonia and was recently reported to downregulate the cell-surface expression of gp130^27^. Treatment with either drug resulted in an obvious reduction of p-STAT3 and IRF9 protein levels (Fig. 2d, left panel) as well as the mRNA levels of the IRDS genes (Fig. 2e), where SC144 (p = 5.4e-5) had a more prominent effect compared to AQ (p = 4.4e-3). Phosphorylated- and total levels of STAT1 were also reduced by the gp130 inhibitors (Fig. 2d, right panel). Together, these data suggest that activation of STAT3 and STAT1, as well as induction of IRF9 and the IRDS genes in HCT116 multicellular spheroids are all under the control of the gp130/JAK signalling pathway.

### STAT3 activation is mediated by a soluble factor

Cytokines that belong to the IL-6 family activate STAT3 through the engagement of gp130^25^. Earlier results have shown that IRF9 and the panel of IRDS genes can be induced not only in MCS but also in confluent cultures of cancer cell lines from multiple origins^14^. We therefore asked whether conditioned medium (CM) from confluent cells could activate STAT3 and induce the expression of IRF9 and the panel of IRDS genes. Indeed, CM from confluent HCT116 cells (72 h in culture) applied for 24 h to freshly seeded non-confluent cells induced phosphorylation of STAT3 as well as STAT1 and STAT2, and also increased protein and mRNA levels of IRF9 (Supplementary Fig. S2a,b). Additionally, the mRNA levels of the IRDS genes were significantly increased by CM (p = 1.6e-9) (Supplementary Fig. S2b). STAT3 phosphorylation was observed already 30 minutes after application of CM, after which it decreases to pre-treatment levels between 2–8 h, increasing again at the 12 h, 16 h and 24 h time points (Supplementary Fig. S2c). The kinetics of STAT3 phosphorylation through gp130 signalling has been reported to display this biphasic pattern^28^. IRF9 protein was readily detected after 12 h of incubation with CM (Supplementary Fig. S2c).

Similarly to what we observed in MCS, inhibiting JAK and gp130 signalling prominently blocked phosphorylation of STAT3 and STAT1 and the expression of IRF9 induced by CM in HCT116 cells (Fig. 2f). mRNA levels of IRF9 and the IRDS panel were also reduced by the JAK- and gp130 inhibitors (Supplementary Fig S2d,e). These results showed that CM from confluent HCT116 cells induced p-STAT3, p-STAT1, IRF9, and the IRDS panel, and that this induction is mediated by gp130/JAK signalling.

The above data suggested that secretion of a cytokine of the IL-6 family may be responsible for the activation of gp130 signalling. Notably, in our previous publication we showed that neither IL-6 nor IL6-R mRNA levels were induced in 3D compared to 2D, and no IL-6 protein could be detected by ELISA in CM from confluent HCT116 cells^14^. In line with this, the use of an IL-6 neutralizing antibody did not affect the level of STAT3 phosphorylation induced by CM in non-confluent cells (Supplementary Fig. S3a). qRT-PCR showed that none of the cytokines of the IL-6 family were significantly up-regulated at the mRNA level in 3D compared to 2D cultures (Supplementary Fig S3b. Note that IL-6 and IL-27 mRNA levels were undetectable). However, OSM mRNA had a mean fold change of 5.9 (p = 0.056), hence, we also analysed the mRNA expression of the OSM-receptor (OSMR) and found it to be significantly induced (p = 0.015, Supplementary Fig. S3b). Furthermore, both OSM and OSMR were upregulated in 3D culture in the microarray from our previous publication (mean fold change of 5.5 and 5.6 respectively)^14^. Despite this, an OSM-neutralizing antibody did not affect the induction of STAT3 phosphorylation or IRF9 protein expression by CM in non-confluent cells (Supplementary Fig. S3c). Thus, it is unlikely that gp130-mediated STAT3 phosphorylation in HCT116 cells in 3D occurs in response to a cytokine from the IL-6 family.

### STAT3 is required for IRF9 and IRDS genes induction in MCS

**Figure 3:**
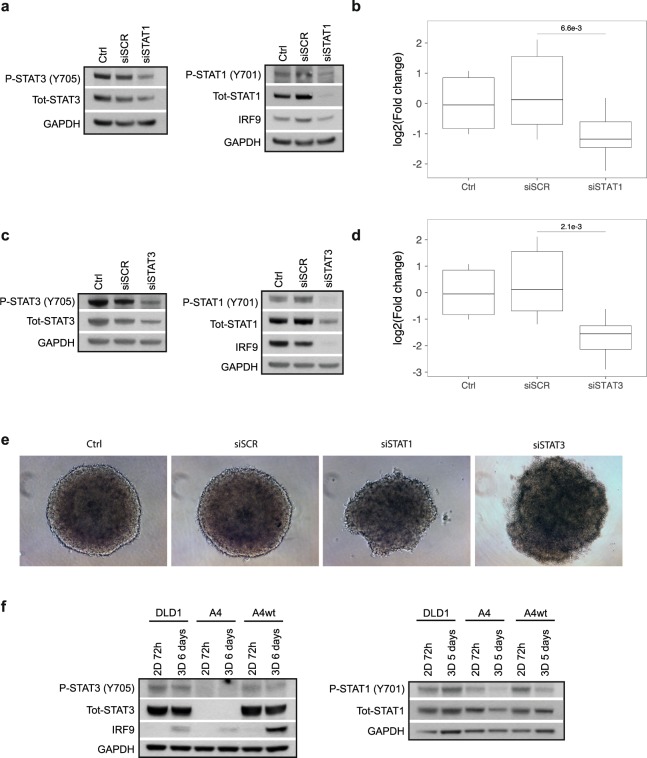
STAT3 is required for IRF9 and IRDS genes induction in MCS. (**a–d**) HCT116 cells were transfected with siRNA and cultured as MCS (3D) for 2 days. (**a**) Western blot analysis showing knockdown of STAT1 and protein expression of STAT3 and IRF9 in the same protein lysates. (**b**) mRNA levels of the IRDS genes, analysed by qRT-PCR (n = 3). mRNA levels of the individual genes are available in Supplementary Fig. S4a. (**c**) Western blot analysis of showing knockdown of STAT3 and protein expression of STAT1 and IRF9 in the same protein lysates. (**d**) mRNA levels of the panel of IRDS genes, analysed by qRT-PCR (n = 3). mRNA expression of the individual genes is displayed in Supplementary Fig. S4b. (**e**) Representative images of HCT116 cells transfected with siRNA and cultured as spheres for 48 h. (**f**) DLD1, A4 and A4wt cells were cultured in 2D or 3D and harvested at the indicated time points. Phosphorylation and expression of STAT1, STAT3 and IRF9 was analysed by Western blot in the same protein lysates. Quantification data of Western blots are available in Supplementary Table 2.

p-STAT1 is known to drive IRF9 expression in response to type I IFNs and also to control ISG transcription as a part of the ISGF3 complex. However, STAT1 knockdown did not affect IRF9 induction in confluent monolayer culture^14^. Efficient knockdown of STAT1 in HCT116 MCS, on the other hand, led to a decrease of IRF9 protein levels (Fig. 3a, right), reduced levels of total- and p-STAT3 (Fig. 3a, left), and significantly (p = 0.0066) decreased the mRNA expression of the IRDS gene panel compared to siRNA control (Fig. 3b, Supplementary Fig. S4a). These data indicate that STAT1 contributes to the induced expression of IRF9 and IRDS genes in MCS.

Our data suggested an up-stream role of STAT3 in the induction of IRF9 in HCT116 MCS. In order to further confirm that STAT3 regulates the expression of IRF9, we used siRNA to knock down STAT3 in MCS and analysed the effects using Western blot and qRT-PCR. Remarkably, a moderate decrease of STAT3 protein levels (Fig. 3c, left panel) was sufficient to abolish the protein expression of IRF9 (Fig. 3c, right panel). Phospho- and total STAT1 levels were also reduced compared to the siRNA control transfected MCS (Fig. 3c, right panel), suggesting that STAT3 regulates both IRF9 and STAT1 expression in this system. Furthermore, the mRNA levels of the IRDS genes were significantly reduced upon STAT3 knockdown compared to siRNA control (Fig. 3d, Supplementary Fig. S4b). However, STAT3 knockdown also altered the morphology of the spheres compared to siRNA control (Fig. 3e). Although we cannot rule out that the observed changes in IRF9 and STAT1 protein levels may depend on the change in sphere morphology, overall, the data indicates that STAT3 regulates IRF9 and STAT1 expression in this system.

In order to further investigate the role of STAT3 in regulating IRF9 expression in MCS we cultured DLD1 sub-lines A4 (STAT3-null) and A4wt (reconstituted with wtSTAT3) as MCS for 6 days. The morphology of the A4 and A4wt spheres differed from DLD1 and appeared rounder and with a defined edge (Supplementary Fig. S4c). IRF9 protein levels were clearly lower in A4 MCS as compared to A4wt, reinforcing the notion that STAT3 plays an important role in IRF9 regulation in MCS (Fig. 3f). Although DLD1 and A4wt showed comparable levels of p-STAT3 in 3D, IRF9 protein expression was higher in A4wt compared to DLD1 (Fig. 3f left panel). This could be due to constitutive STAT3 expression from an exogenous promoter in the A4wt cells. Interestingly, p-STAT1 levels were decreased in 3D compared to 2D culture in A4wt, indicating that p-STAT1 is regulated differently in these cells compared to the parental DLD1 and does not contribute to IRF9 induction (Fig. 3f right panel). Together, these data suggest that STAT3 is an important regulator of IRF9 expression in MCS.

### STAT3 is recruited to the IRF9 promoter in MCS

**Figure 4:**
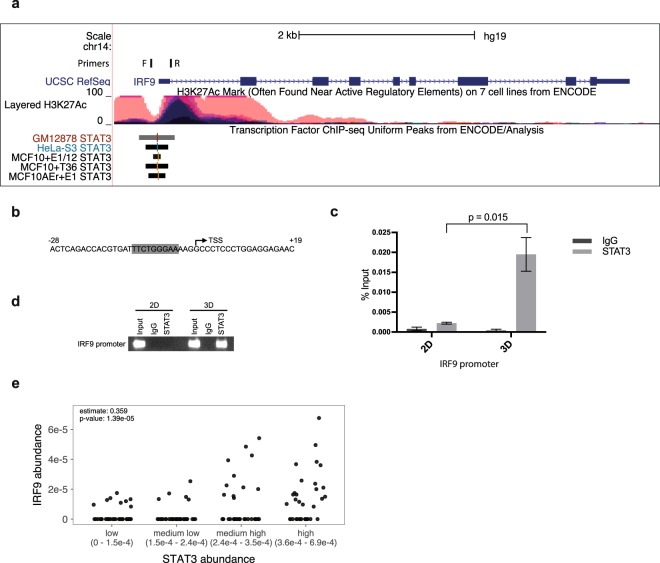
STAT3 is recruited to the IRF9 promoter in cells grown in 3D. (**a**) A schematic depiction of CHIP-seq data from the UCSC genome browser showing enrichment of STAT3 at the IRF9 promoter region (UCSC genome browser assembly February 2009 GRch37/hg19). (**b**) The position and sequence of the STAT binding site in the IRF9 promoter region (RefSeq: NM_006084.4). (**c**,**d**) CHIP assay results evaluating the binding of STAT3 to the IRF9 promoter in HCT116 cells cultured in 2D and 3D. Chromatin was immunoprecipitated with an anti-STAT3 antibody followed by qRT-PCR (n = 3) (**c**) or semi-quantitative PCR (**d**). Location of the primers used are shown in figure (**a**). Error bars represent S.E.M. (**e**) Correlation analysis between STAT3 and IRF9 protein abundance in primary colorectal cancer tumour samples (n = 95) and cell lines (n = 44). The x-axis is binned in four groups (n = 34–35 per group) according to abundance of STAT3. Scatter plot is shown with Pearson correlation coefficient and corresponding p-value.

Next, we addressed the question whether STAT3 directly regulates the expression of IRF9 in MCS. Chromatin immunoprecipitation (ChIP) sequencing data from the University of California, Santa Cruz (UCSC) genome browser indicated that STAT3 binds to the IRF9 promoter near the transcription start site in multiple cancer cell lines (Fig. 4a). Closer investigation of the sequence revealed a STAT consensus binding sequence (TTCNNNGAA, Fig. 4b). In order to determine if STAT3 binds in the IRF9 promoter in MCS, we performed ChIP with an anti-STAT3 antibody in HCT116 cells cultured in either 2D for 24 h or 3D for 6 days. Immunoprecipitated DNA was analysed by qRT-PCR or PCR using primers spanning the potential STAT3-binding site in the IRF9 promoter (location of primers depicted in Fig. 4a). Increased enrichment of STAT3 at the IRF9 promoter was observed in spheres compared to non-confluent cells cultured in 2D using either qRT-PCR (Fig. 4c, p = 0.015) or semi-quantitative PCR (Fig. 4d). c-FOS, a well established STAT3-activated gene, was used as a positive control for the STAT3 activation in MCS, and the data showed a significant enrichment (p = 0.04) of STAT3 in 3D compared to 2D at this promoter (Supplementary Fig. S5). These results show that STAT3 is enriched at the IRF9 promoter in HCT116 MCS, suggesting that STAT3 directly drives IRF9 transcription in this system.

In order to further investigate the relationship between STAT3 and IRF9 protein expression in a broader context we analysed publicly available proteomics data on 95 primary tumour samples from 90 colorectal cancer patients and 44 colorectal cancer cell lines^29^. We found a moderate, but significant (r = 0.359, p = 1.39e-5), positive correlation between STAT3 and IRF9 protein abundance (Fig. 4e). Although this data does not implicate a causal relationship, it further suggests a connection between STAT3 and IRF9 expression in colorectal cancer.

## Discussion

In this study we show that STAT3 is activated in MCS of HCT116 cells through gp130-JAK signalling. STAT3 directly binds to the IRF9 promoter suggesting that STAT3 activation leads to the induction of IRF9 transcription. Elevated levels of IRF9 in turn contribute to the expression of IRDS genes, which has previously been shown to correlate with DNA damage resistance. We also report a positive correlation between STAT3 and IRF9 protein abundance in primary colorectal tumour samples and cell lines.

Abnormal activation of STAT3 is a common event in many types of malignancies. High levels of phosphorylated STAT3 are unfavourable for overall survival and can be correlated to lymph node metastasis in colorectal cancer patients^30^. Several publications have also shown that high cell density results in maintained STAT3 activation in cancer cell lines of different origin^18,19,31,32^ and that this occurs in a ligand-independent manner^18,19^. Here we show that STAT3 is activated in the HCT116 colorectal cancer cell line cultured as MCS, where cells grow at high density in 3D suspension structures. In accordance with previous reports, we found that this activation was effectively blocked by JAK inhibitors^19,31,33^. However, transferring supernatant from a confluent culture to non-confluent cells also resulted in activation of STAT3, indicating that the mechanism in our system could be ligand-dependent. Additionally, inhibiting the activity of the receptor subunit gp130 prevented STAT3 activation in spheres or in CM-treated 2D cultures, which strengthens the hypothesis that a cytokine that acts through gp130 may be involved. We previously reported that mRNA expression of IFNs could not be detected in MCS, nor secretion of IFNs in the CM from MCS or confluent cells by ELISA, thus excluding IFNs as the responsible cytokine(s)^14^. On the other hand, among the IL-6 family of cytokines, LIF, CT-1, IL-11RA, OSM, and OSMR were upregulated in 3D compared to 2D (with a cut-off of 2-fold induction) in the microarray from our previous publication^14^. Therefore, we used qRT-PCR to validate the mRNA expression of all the 8 members of the IL-6 family of cytokines in HCT116 cells grown in 3D compared to 2D cultures. Although none of the cytokines were significantly upregulated on the mRNA level, we decided to further investigate the possible involvement of OSM, which had a mean fold change of 5.9 (p = 0.056) in 3D. However, our experiments showed that OSM is not responsible for the induction of pSTAT3 or IRF9 by CM in HCT116 cells.

3D cell culturing systems, compared to 2D, more accurately recapitulate the *in vivo* biochemical and physical features of solid tumours. For example, cells in the core are exposed to hypoxia and nutrient deprivation, resulting in a quiescent as well as apoptosis resistant phenotype^34,35^. MCS have also been shown to be more resistant to chemotherapeutic drugs^36,37^. Park *et al*. investigated 100 different cancer cell lines cultured as MCS and classified them into four groups based on morphology; round, mass, aggregate and none (those who did not form spheres). They found that JAK-STAT signalling was one of the pathways correlated to the round-type sphere, where STAT3 expression and activation was higher compared to the mass- or aggregate-type spheres^33^. Furthermore, the authors found that STAT3 affects tumour permeability and drug sensitivity in MCS. Blocking STAT3 activity in round-type spheres using the JAK-inhibitor AG490 resulted in loosening of the outer layer of the sphere and decreased hypoxia in the core. Additionally, the JAK-inhibitor improved drug penetration and increased sensitivity to other chemotherapeutic drugs^33^. We did not observe this change in morphology in HCT116 spheres upon treatment with JAK- or gp130-inhibitors (data not shown), despite effective inhibition of STAT3 phosphorylation. On the other hand, reducing total STAT3 levels using RNAi resulted in an obvious morphological change into an aggregate, suggesting that un-phosphorylated STAT3 may be involved in the formation of the round-type sphere in HCT116 cells.

It has been established that IRF9 expression can be induced by IL-6, although not directly by STAT3^16^. IFNγ signalling activates STAT1 and is a well-known inducer of IRF9 transcription. However, STAT1 was not directly involved in the activation of the IRF9 promoter by IFNγ^17^. Using RNAi to knockdown STAT1 in HCT116 cells and U3A, a STAT1-null sub-line of fibrosarcoma cell line 2fTGH, we previously showed that STAT1 was not required for, but augmented, the expression of IRF9 and IRDS genes induced by high cellular density^14^. In this study we observed activation of both STAT1 and STAT3 in HCT116 MCS, which prompted us to further investigate which transcription factor was responsible for the IRF9 induction in this system. Since JAK and gp130 inhibitors abolished both STAT3 and STAT1 activation, either can be responsible for IRF9 induction. Reducing STAT3 levels by RNAi in HCT116 MCS completely abolished IRF9 protein expression strongly suggesting that STAT3 drives IRF9 expression. On the other hand, STAT3 knockdown changed the morphology of the spheres which, as discussed above, could affect the induction of JAK-STAT signalling and, therefore, IRF9 expression. STAT1 knockdown also affected IRF9 expression and even the morphology of the sphere. Although these effects were less pronounced compared to STAT3, it nevertheless points at the involvement of STAT1 in this phenotype. Furthermore, knocking down IRF9 in MCS clearly reduced total- and phosphorylated levels of STAT1 suggesting the existence of a positive feedback loop between STAT1 and IRF9. In contrast, the levels of STAT3 remained unaffected in cells with knockdown of IRF9, suggesting that STAT3 is upstream in the signalling pathway leading to the induction of IRF9. Indeed, absence of STAT3 clearly reduced the induction of IRF9 in MCS of STAT3-null A4 cells as compared to the parental DLD1, while STAT3 reconstitution in A4wt cells led to a prominent increase of IRF9 levels in MCS. Interestingly, p-STAT1 levels were reduced in both A4 and A4wt MCS compared to monolayer culture, suggesting a secondary role of STAT1 in the IRF9 induction in MCS. This data reinforced the notion of STAT3 as an important regulator of IRF9 induction in MCS.

ChIP-seq data from the UCSC genome browser indicated that STAT3 binds near the transcriptional start site in the IRF9 promoter in multiple cell lines. Notably, a recent publication including ChIP-seq data from two diffuse large B-cell lymphoma cell lines shows STAT3 enrichment at this location in the IRF9 promoter, although, in this case STAT3 appeared to negatively regulate IRF9 transcription^38^. We found that STAT3 was significantly enriched at the IRF9 promoter in HCT116 cells grown as MCS compared to non-confluent cells in 2D. Considering the increase in IRF9 mRNA and protein levels in MCS, our data strongly suggest that STAT3 directly drives IRF9 transcription in this model. We investigated the sequence near the transcription start site in the IRF9 promoter in search of a possible STAT3 binding site and found the sequence TTCTGGGAA at position −12. Interestingly, the same sequence has been identified as the acute-phase response element (APRE) in the rat α2-macroglobulin promoter, which STAT3 binds to in response to IL-6^39,40^.

In summary, our data indicates a connection between STAT3 and IRF9 in colorectal cancer. We propose a novel mechanism where STAT3 is activated, through gp130 signalling, in multicellular spheroids resulting in increased expression of IRF9. This, in turn, leads to expression of IRDS genes, underlining a mechanism by which drug resistance is regulated in tumours.

## Materials and Methods

### Cell lines and reagents

HCT116 and DLD1 colorectal carcinoma cell lines were obtained from ATCC. Sub-lines of DLD1, A4 (with homozygously deleted STAT3) and A4wt (A4 reconstituted with the wild-type variant of STAT3)^41^ were a gift of Zhenghe Wang, Genetics and Case Comprehensive Cancer Center, Case Western Reserve University. HCT116 cells were cultured in RPMI 1640 (Gibco), DLD1, A4 and A4wt in DMEM/High Modified (HyClone). Culture media was supplemented with 10% fetal bovine serum, 2mM L-glutamine, 100 μg/ml streptomycin and 100 U/ml penicillin. All cell lines were verified by ATCC using short tandem repeat analysis. The cells were regularly tested for mycoplasma using the Mycoplasma Detection kit (Jena Bioscience). Ruxolitinib was purchased from InvivoGen, Pyridone 6 (JAK inhibitor I) from Merck Chemicals and Life Science AB, SC144 hydrochloride from Sigma-Aldrich and Atovaquone from Selleckchem.

### Generation of multicellular spheroids and condition medium

MCS of HCT116, A4 and A4wt were generated using the “hanging drop” technique, as described previously^14^. DLD1 cells were seeded 10 000 cells/well in 200 μl media in an ultra-low attachment 96-well plate (Sigma-Aldrich, CLS7007) followed by centrifugation at 1500 rpm for 15 min. The MCS were incubated for 6 days before analysis (unless otherwise stated). This time point was used to allow the spheres to reach a diameter of at least 500 μm, since MCS of this size have been shown to mimic the *in vivo* conditions of small solid tumours^42^.

Condition medium (CM) was collected from confluent monolayer cells (cultured for 72 h), centrifuged to remove cell debris and then stored at −20 °C until further use.

### Western blot

Cell pellets were lysed in a modified RIPA buffer (50 mM Tris-HCl pH7.4, 150 nM NaCl, 1 mM EDTA, 1% NP-40 and 1% Glycerol) supplemented with protease inhibitor cocktail cOmplete and phosphatase inhibitor phosSTOP (Roche), incubated 20 min on ice and then centrifuged at 18000 rpm for 20 min to remove cell debris. Protein concentration was determined using Bradford assay (Bio-Rad Laboratories). A total of 20–40 μg of protein was separated on 4–12% Bis-Tris gels (NuPAGE, Life Technologies). Transfer was done using the iBlot system (Thermo Fisher Scientific) and the nitrocellulose membranes were blocked in either 5% Blotting grade blocker (BioRad) or 5% BSA (Medicago) in TBS supplemented with 0.1% Tween-20 (Merck) for 1 h. Membranes were incubated with primary antibodies diluted in blocking agent over night at 4 °C and then for 1 h with secondary antibodies at room temperature (HRP-conjugated anti-rabbit from Cell Signaling Technology, #7074). The proteins were detected using Western Lightning Plus-ECL (PerkinElmer) and captured using Kodak M35 X-omat processor. Quantification of Western blots was performed using Adobe Photoshop CS6 version 13.0 x64. Band intensity was normalized to that of GAPDH. Quantification data is available in Supplementary Table 2. The following antibodies were from Cell Signaling Technology: IRF9 (#76684), STAT1 (#9172), P-Y701-STAT1 (#9171), STAT2 (#72604), P-Y690-STAT2 (#88410), STAT3 (#4904), P-Y705-STAT3 (#9145). GAPDH antibody (ab9485) was purchased from Abcam.

### RNA interference

Cells were seeded 2 × 10^5^ cells/well in 6-well plates and cultured at standard conditions for 24 h. The cells were transfected in antibiotic-free media with 30 nM small interfering RNA (siRNA) using Lipofectamine 2000 according to the manufacturer’s instructions. After 24 h the cells were trypsinised, counted and re-seeded as spheres (as described above). 48 h later the spheres were harvested, and knockdown efficacy was assessed by Western blot and qRT-PCR. siRNA targeting IRF9 was purchased from Dharmacon (ON-TARGET plus Human siRNA smart pool). STAT3 siRNA was purchased from MWG Operon Eurofins; 5′- GCAACAGAUUGCCUGCAUU-3′. STAT1 siRNA was purchased from Integrated DNA technologies (IDT); 5′- ACUCAAGAAGAUGUAUUU-3′. Initial experiments were performed with additional siRNAs targeting STAT1 and STAT3, with similar results (sequences are available in Supplementary Table S1). A Blast search showed 0% identity overlap between STAT1 siRNA with STAT3 and vice versa.

### Electrophoretic mobility shift assay

Nuclear extracts, probe labelling and EMSA were performed as previously described with some modifications^43^. Nuclear extracts were obtained as follows: cells were washed in PBS, and cell pellets were resuspended in an ice-cold buffer (20 mM HEPES pH 7.9, 10 mM KCl, 0.1 mM EDTA, 0.1 mM EGTA, 2.5 mM DTT, 0.2% NP40) and left for 20 minutes on ice. The nuclei pellet was collected by centrifugation and resuspended in ice-cold buffer (20 mM HEPES pH7.9, 25% Glycerol, 0.4 M KCl, 1 mM EDTA, 1 mM EGTA, 2.5 mM DTT). The tubes were vortexed vigorously at 4 °C for 15 minutes, centrifuged at high speed for 10 minutes at 4 °C and the supernatant containing nuclear proteins was collected and stored in aliquots at –70 °C. All buffers contained protease and phosphatase inhibitors from Roche according to their instructions. The hSIE probe (5′GTCGACATTTCCCGTAAATC3′) was end-labelled using γ-^32^p-ATP (Perkin Elmer) and T4 Polynucleotide kinase (New England Biolabs) according to manufacturer’s instructions. For EMSA, 10 µg of nuclear extracts were pre-incubated for 5 minutes in binding buffer containing 25 mM HEPES pH 7.9, 10% glycerol, 5 mM DTT, 150 mM KCl and 2 µg/ml polydI:dC (Amersham Pharmacia) followed by the addition of the labelled probe and further incubation for 30 minutes at RT. The complexes were separated on a 4% non-denaturing polyacrylamide gel in Tris/Glycine/EDTA buffer (40 mM Tris-base, 200 mM Glycine, 0.1 mM EDTA). Gels were air-dried and visualized by autoradiography.

### Quantitative real-time PCR

RNA was extracted using the RNA NucleoSpin II kit (Macherey-Nagel) and treated with DNase (Ambion Turbo DNA-free, Life Technologies). 500 ng of RNA was used to generate cDNA using First Strand cDNA Synthesis kit (K1612, Invitrogen by Thermo Fisher Scientific) according to the manufacturer’s instructions. qRT-PCR was performed using PowerUp SYBR Green Master Mix (A25777, Thermo Fisher Scientific) on the CFX96 Touch Real-Time PCR Detection System (Bio-Rad) at the following conditions: 50 **°**C for 2 min, 95 **°**C for 2 min followed by 40 cycles of 95 **°**C for 3 sec and 60 **°**C for 30 sec, finishing at 65 **°**C for 5 sec. Expression was normalized to the mean of two internal controls; U48 and B2M and the fold gene expression was calculated using the 2^−ΔΔCT^-method^44^. The corresponding primers for each target are specified in Supplementary Table S1.

### Chromatin Immunoprecipitation (ChIP)

Spheres were seeded as described previously^14^ and cultured for 6 days before analysis. Non-confluent adherent cells were seeded 24 h before analysis in 10 cm dishes, 1.5 × 10^6^ cells/dish and were used as controls. Cells were cross-linked with 1% formaldehyde for 15 min, 2D cells were rocked gently and spheres were rotated, followed by quenching in 125 mM glycine for 5 min. The 2D cells were harvested using a cell scraper and all cells were lysed in 10 ml cell lysis buffer (5 mM PIPES, 85 mM KCl and 0.5% Nonidet P-40) and then in 400 μl nuclei lysis buffer (50 mM Tris-HCl pH 8, 10 mM KCl and 1% SDS). Protein concentration was determined using Pierce BCA protein Assay Kit (Thermo Scientific) and the samples were diluted in nuclei lysis buffer to achieve equal protein concentration in all samples. Cells were soincated using a Bioruptur Sonicator (Diagenode). Samples were pre-cleared by adding Salmon sperm DNA/Agarose A beads (30 µl/ml) (Merck) and rotated for 1 h at 4 **°**C. 5 μg of STAT3 antibody (sc-482, Santa Cruz Biotechnology) or IgG Rabbit (PB644, Merck) was added to the samples and rotated over night at 4 **°**C. Salmon sperm DNA/Agarose A beads were used to pull down the antibody and DNA was eluted in elution buffer (1% SDS, 0.1 M NaHCO3). Cross-linking was reversed by adding NaCl to a final concentration of 200 mM and incubating the samples overnight at 65 **°**C. The samples were treated with RNase-A (Thermo Fisher Scientific) and proteinase K (Finnzymes, F-202S) and the DNA was isolated using the QIAquick PCR purification kit (Qiagen) according to the manufacturer’s instructions. qRT-PCR was performed using the KAPA SYBR FAST qPCR Master mix (KAPA Biosystems) at the following conditions: 95 **°**C for 3 min, followed by 40 cycles of 95 **°**C for 3 sec and 60 **°**C for 30 sec, finishing at 65 **°**C for 5 sec. The corresponding primers for each target are specified in Supplementary Table S1.

### Semi-quantitative PCR

2 μl of the ChIP product was amplified by PCR using the KAPA2G HotStart ReadyMix PCR kit (KAPA Biosystems) and run according to the manufacturer’s instructions. The following cycling conditions were used: 95 **°**C for 3 min, 35 cycles of 95 **°**C for 10 sec, 60 **°**C for 10 sec, 72 **°**C for 1 sec, followed by a final extension at 72 **°**C for 1 min. 3 μl of the PCR product was run on a 1.5% agarose gels, stained with SYBR® Safe DNA gel stain (Invitrogen) and image captured with the Gel Doc EZ System (BioRad). The corresponding primers for each target are specified in Supplementary Table S1.

### Immunohistochemistry

MCS were fixed in 2% buffered formalin overnight, dehydrated, embedded in paraffin and sectioned. Sections were deparaffinized with xylene, boiled in either citrate buffer (IRF9 and STAT3) or EDTA (p-STAT3) and incubated in 0.5% hydrogen peroxide followed by blocking in 5% milk. Primary antibodies were incubated overnight, IRF9 (ab56677) from abcam, P-Y705-STAT3 (#4113) and STAT3 (#9139) from Cell Signaling Technology. Biotinylated secondary anti-mouse antibody was applied for 30 min at room temperature. The specific signal was detected using ABC Vectastain and DAB peroxidase, both from Vector Laboratories. Finally, the sections were counterstained with Mayer’s haematoxylin.

### Neutralizing antibody

Cells were seeded 2 × 10^5^ cells/well in 6-well plates and cultured at standard conditions for 24 h. The cells were washed with PBS before adding new fresh media, conditioned media or media containing IL-6 (5 ng/ml) and IL-6R (10 ng/ml) or OSM (5 ng/ml), immediately followed by addition of IL-6 neutralizing antibody (Peprotech, 500-P26G), OSM neutralizing antibody (Thermo Fisher Scientific, PA5–47002), or goat IgG control antibodies (Sigma-Aldrich, I5256). After 1 h the cells were harvested and the effect of the neutralizing antibodies on tyrosine phosphorylation of STAT3 and IRF9 expression was analysed by Western blot.

### Statistical analysis

Statistical analysis was performed in the R statistical programming language^45^ and the GraphPad Prism (for Chip data analyses, version 7.0, GraphPad Software, www.graphpad.com), with the help of the ggplot2 package^46^ for visualization. A significance threshold of 0.05 was utilized throughout the study. Statistical analysis was performed using the independent t-test (two-sided, equal variance) unless otherwise stated. Correlation analysis was performed using the normalized (abundance/total sample abundance) abundance values of the respective proteins with the null-hypothesis that the Pearson’s correlation coefficient is equal to 0.

In cases where the mRNA expression of the IRDS genes (OAS1, IFI6, IFI27 and IFI44) was evaluated, experiments were performed in biological triplicate, unless otherwise stated, and results for each gene were pooled into the stated conditions previous to hypothesis testing. The lower and upper hinges in the box plots correspond to the first and third quartiles. Whiskers extend to the largest or smallest value within 1.5 * interquartile range (IQR), outliers are represented as asterisks.

## Supplementary information

Supplementary Information

Supplementary Table 2

## Data Availability

The datasets generated during the current study are available from the corresponding author on request. Data originating from outside of this study are appropriately referenced and publicly available from that source.
