# Supplementary material for: STAT3 is activated in multicellular spheroids of colon carcinoma cells and mediates expression of IRF9 and interferon stimulated genes
Source: Sci Rep. 2019 Jan 24;9:536. doi: 10.1038/s41598-018-37294-z (PMC6345781; doi:10.1038/s41598-018-37294-z)
Supplement: Supplementary file 1 — Supplementary Information [file 41598_2018_37294_MOESM1_ESM.pdf]

# STAT3 is activated in multicellular spheroids of colon carcinoma cells and mediates expression of IRF9 and interferon stimulated genes

Elin Edsbäcker[1,2*], Jason T. Serviss[1], Iryna Kolosenko[1,2], Caroline Palm-Apergi[2], Angelo De Milito[1] and Katja Pokrovskaja Tamm[1]

[1] Department of Oncology-Pathology, Cancer Center Karolinska, Karolinska Institutet, Stockholm, Sweden

[2] Department of Laboratory Medicine, Clinical Research Center, Karolinska Institutet, Stockholm, Sweden

***Corresponding author**: Elin Edsbäcker, Cancer Center Karolinska, Department of Oncology-Pathology, Karolinska Institutet, 17176 Stockholm, Sweden; Tel.:+46851775431; Fax: +468339031; E-mail: elin.edsbacker@ki.se

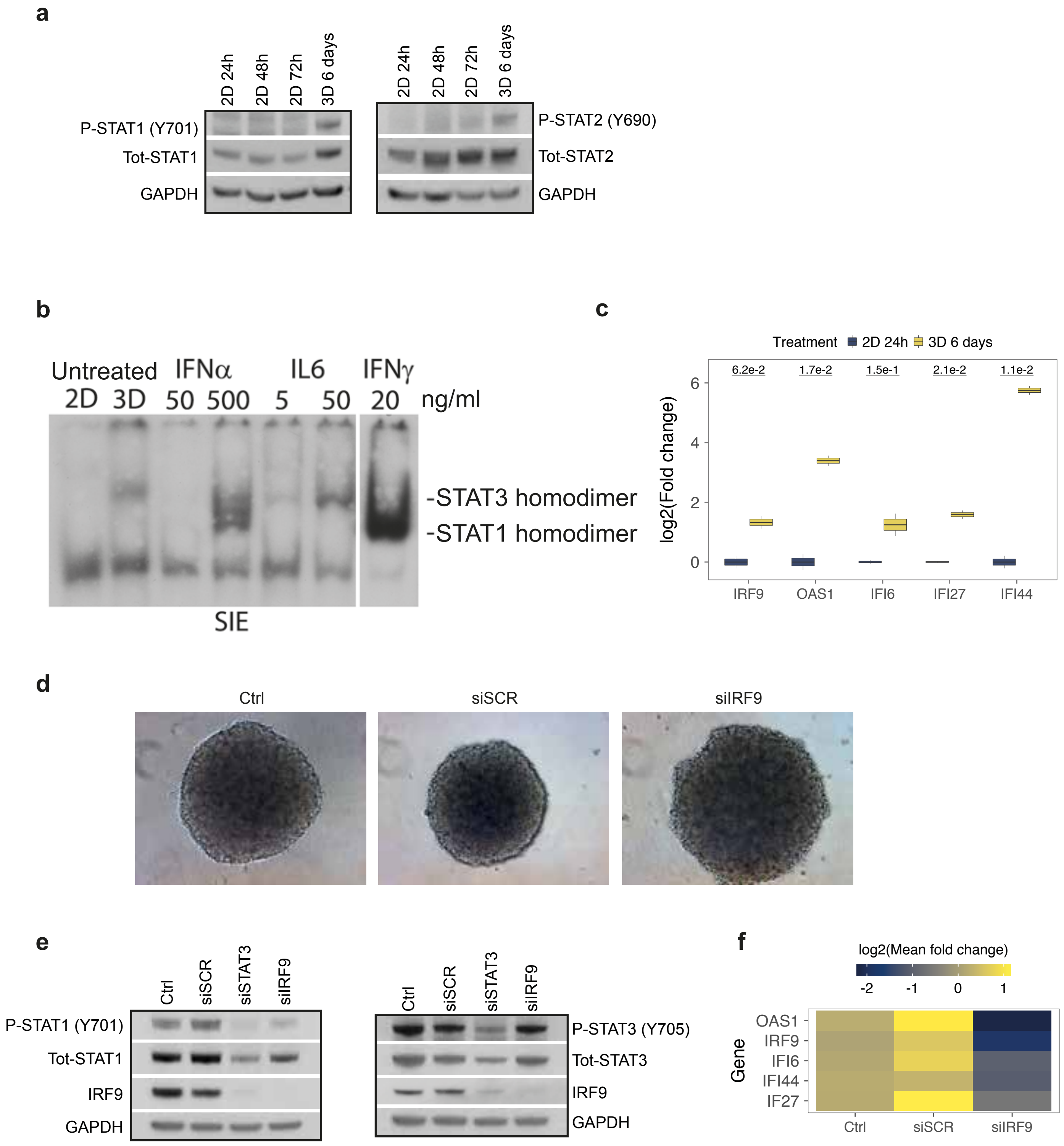


**Supplementary Figure S1. Activation of STAT1/STAT2 and induction of IRF9 and IRDS genes in MCS** (a) HCT116 cells cultured as MCS compared to cells in monolayer harvested at indicated time points was subjected to Western blot analysis of tyrosine-phosphorylation and total expression of STAT1 and STAT2. (b) EMSA was performed on the nuclear cell extracts from HCT116 cells cultured as MCS (3D) compared to cells in monolayer (2D) treated with IFNα, IL6 or IFNγ at indicated concentrations. (c) DLD1 cells were cultured in 2D and 3D and harvested at indicated time points. mRNA expression of IRF9 and the indicated ISGs was analysed using qRT-PCR (n=2). (d) Representative images of control or siRNA transfected HCT116 cells cultured as MCS for 48h. (e) Full length blots of cropped membranes displayed in Fig. 1f. (f) Cells were transfected with siRNA targeting IRF9 and cultured as MCS for 48h. Heatmap showing the mean mRNA expression of the indicated genes (n=3). Quantification data of Western blots are available in Supplementary Table 2.

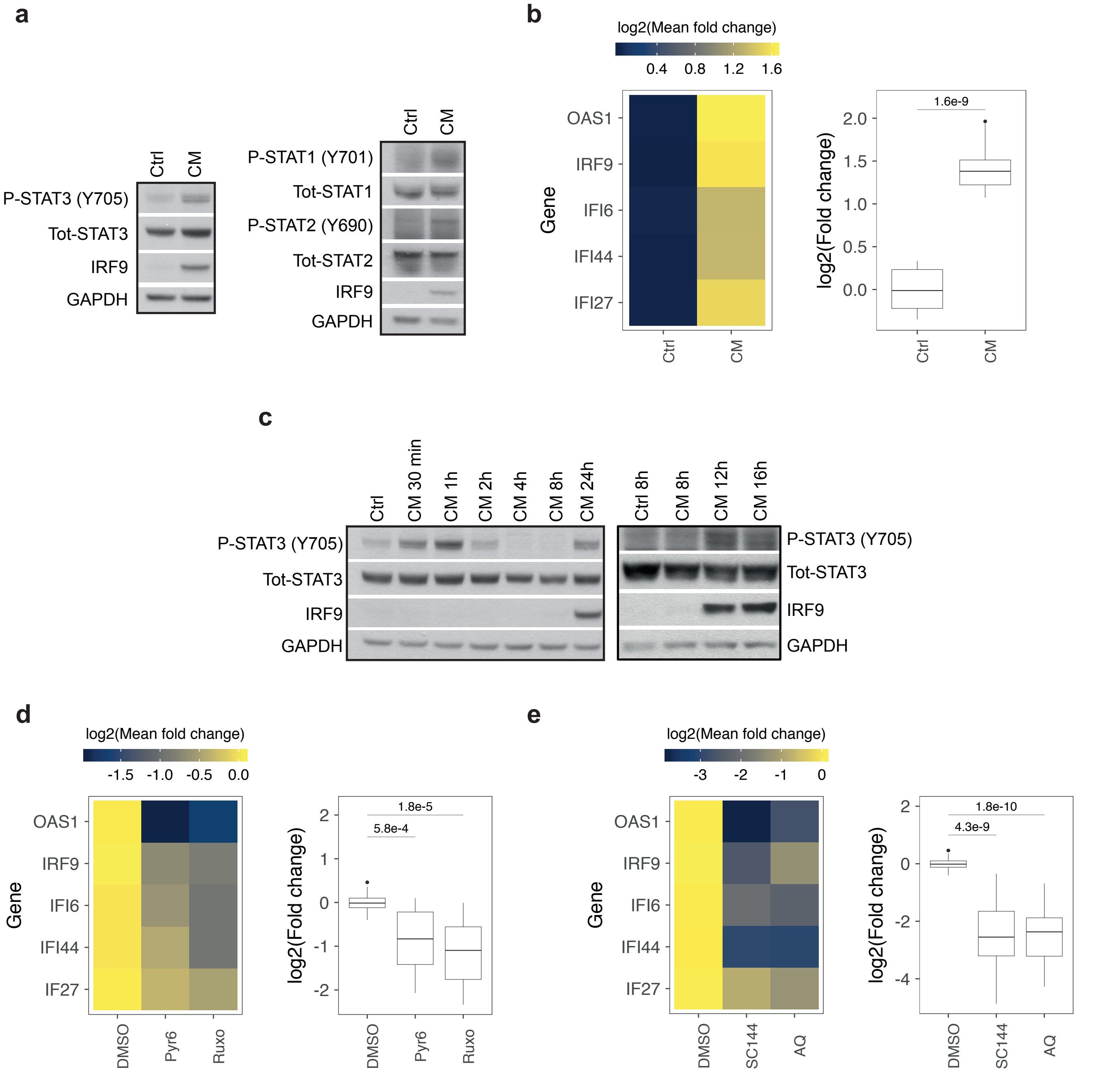


**Supplementary Figure S2. Activation of STAT1/STAT3 and induction of IRF9 and IRDS genes by condition media** (a, b) CM from confluent HCT116 cells was applied to freshly seeded non-confluent cells and incubated for 24h. (a) Phosphorylation and total protein expression of STAT1, STAT2, STAT3 and IRF9 was analysed by Western blot. (b) qRT-PCR analysis of mRNA expression of the indicated ISGs (n=3). The heatmap displays mean mRNA expression of the individual genes and the box plot show mRNA levels of the IRDS panel as a group. (c) CM was applied to freshly seeded non-confluent cells, harvested at indicated time points and subjected to Western blot analysis of STAT3 tyrosine-phosphorylation and IRF9 protein expression. (d, e) HCT116 cells seeded at low density were treated with CM and JAK or gp130 inhibitors for 24h. mRNA expression of IRF9 and the IRDS genes in cells treated with JAK inhibitors (d) or gp130 inhibitors (e) were analysed by qRT-PCR (n=3). Heatmaps show mean mRNA expression of the indicated genes, box plots display the mRNA levels of the IRDS panel as a group. Quantification data of Western blots are available in Supplementary Table 2.

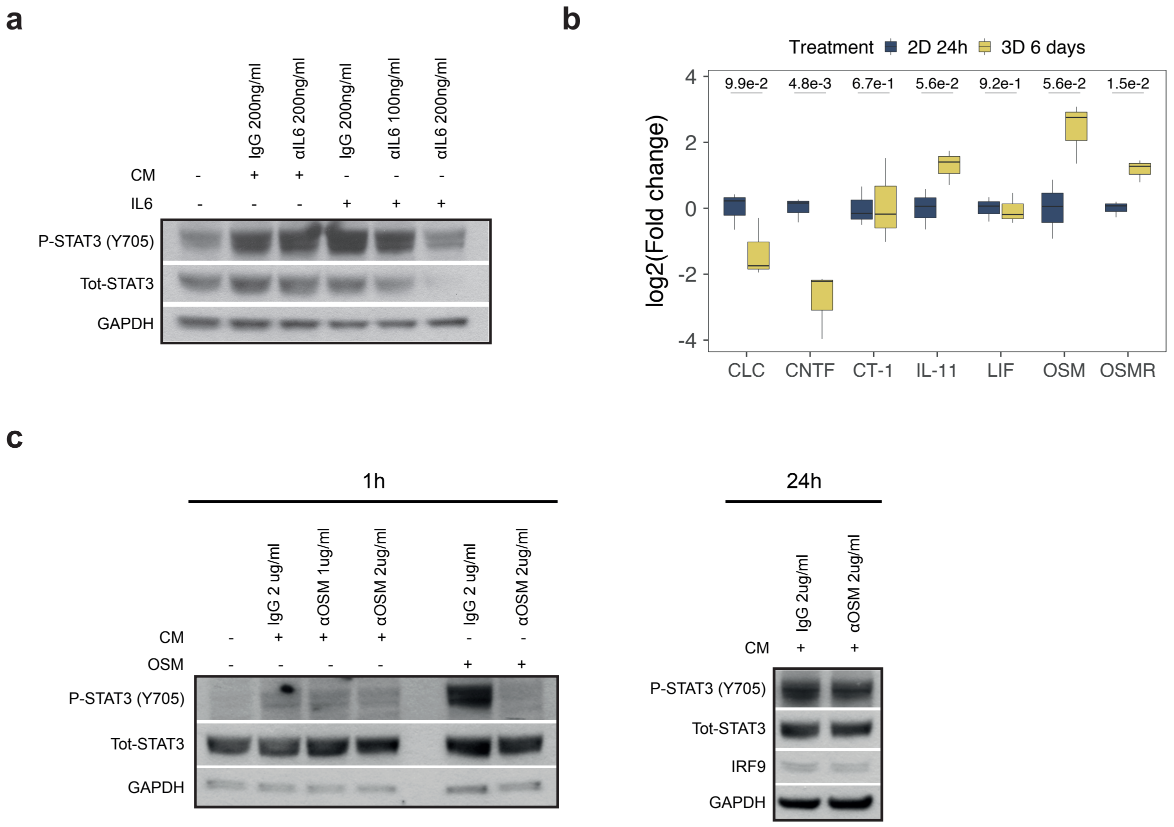


**Supplementary Figure S3. The IL-6 family of cytokines in MCS and CM** (a) Western blot analysis of STAT3 phosphorylation following 1h incubation with CM or IL6 (5ng/ml) and IL6R (10ng/ml) in the presence of neutralizing anti-IL6 antibody at indicated concentrations. (b) HCT116 cells cultured as MCS (3D) compared to cells in monolayer (2D) harvested at indicated time points was subjected to qRT-PCR analysis of mRNA levels of the indicated genes (n=3). (c) Western blot analysis of STAT3 phosphorylation and IRF9 expression following 1h or 24h incubation with CM or OSM (5ng/ml) in the presence of neutralizing anti-OSM antibody at indicated concentrations. Quantification data of Western blots are available in Supplementary Table 2.

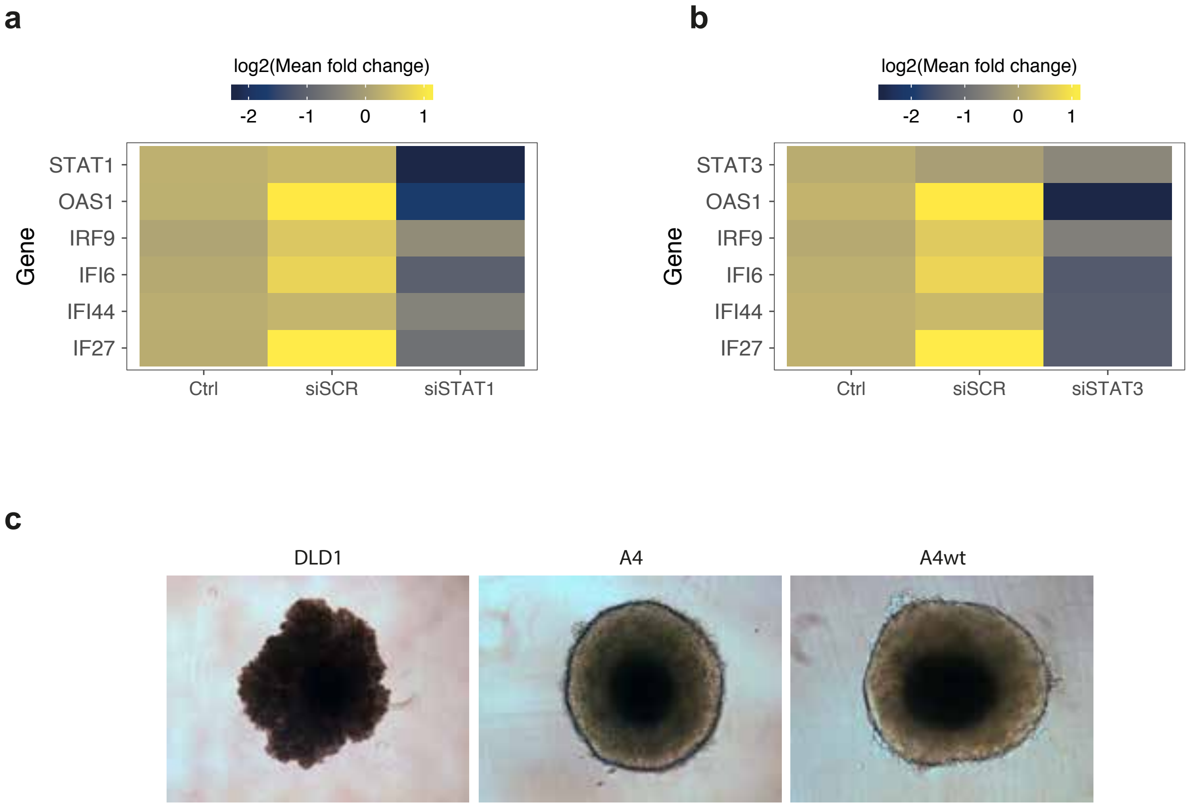


**Supplementary Figure S4. Effect of STAT1 or STAT3 knockdown on IRDS gene expression in MCS and images of DLD1, A4 and A4wt MCS** (a, b) HCT116 cells were transfected with siRNA and cultured as MCS (3D) for 2 days. Heatmap is showing the mean mRNA levels of the indicated IRDS genes and STAT1 (a) or STAT3 (b), measured by qRT-PCR (n=3). (c) Representative images of DLD1, A4 and A4wt cells grown as MCS for 6 days.

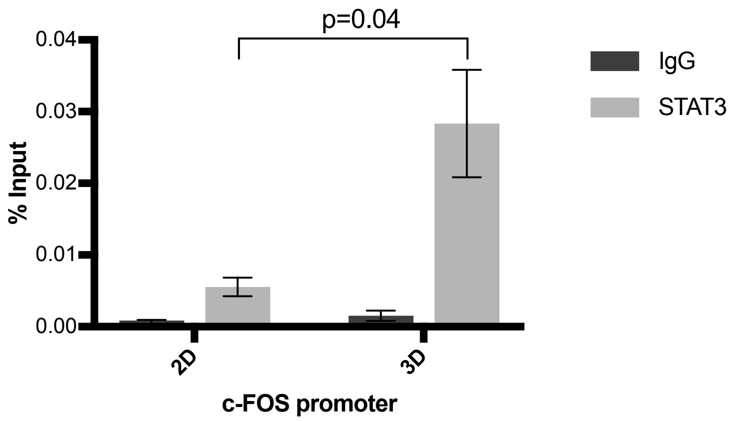


**Supplementary Figure S5. STAT3 is enriched at the c-FOS promoter in MCS.** CHIP assay was performed to determine the binding of STAT3 to the c-FOS promoter on cells cultured in 2D compared to 3D and analysed by qRT-PCR (n=3). Error bars represent S.E.M.

**Supplementary table S1. Sequences of primers and siRNA**

siRNA
From IDT:
STAT1 5'-CUUGACAGUAAAGUCAGAAAU-3'
From Eurofins MWG Operon:
STAT3 5'- AACAUCUGCCUAGAUCGGCUA-3'

Primers used for qRT-PCR
From IDT:
IRF9-F 5´-CATGGCTCTCTTCCCAGAAA-3´
IRF9-R 5´-AGCTCTTCAGAACCGCCTAC-3´
STAT1-F 5´-TGAATATTCCCCGACTGAGC-3´
STAT1-R 5´-AGGAAGACCCAATCCAGATGT-3´
IFI6-F 5´-AGCAGCGTCGTCATAGGTAATA-3´
IFI6-R 5´- CTACTCCTCATCCTCCTCACTATC-3´
IFI44-F 5´-TGTAACGCATCAGGCTTTGG-3´
IFI44-R 5´-CGGCAGGTATTTGCCATCTT-3´
U48-F 5´-AGTGATGATGACCCCAGGTA-3´
U48-R 5´-GGTCAGAGCGCTGCGGTGAT-3´
IL6-F 5´-CAGCTATGAACTCCTTCTCCAC-3´
IL6-R 5´-CTGGCTTGTTCCTCACTACTC-3´
IL11-F 5´-GAGAGGCTTGCTTGGGATATAG-3´
IL11-R 5´-CTTTGACCTGGAGACAGTCATT-3´
IL27-F 5´-TCCTTGGAGCTCGTCTTATCT-3´
IL27-R 5´GGCTTTCAGTTACTGGGTAGAG--3´
CT1-F 5´-GTGTCTGTCTGTCTGCTCTTAG-3´
CT1-R´-CTGAAGGAAAGGAGGACATCAA-3´
CNTF-F 5´-CCTGGGACAGTTGAGTTAAGG-3´
CNTF-R 5´-CGACTTGGAGAAGAAGGGTATG-3´
LIF-F 5´-TGTCGCTCTCTAAGCACTTTA-3´
LIF-R 5´-GGTCCACACCAGCAGATAAA-3´
CLC-F 5´-GCTTCTGACTTCTGACCTTCTC-3´
CLC-R 5´-AATCCTGTGGTGTGGCTATG-3´
OSM (PrimeTime® Assay ID: Hs.PT.58.45339180)
OSMR (PrimeTime® Assay ID: Hs.PT.58.4051301)
B2M (PrimeTime® Assay ID: Hs.PT.58v.18759587)

From Eurofins MWG Operon:
STAT3-F 5´-GAGAAGGACATCAGCGGTAAG-3’
STAT3-R 5´-CAGTGGAGACACCAGGATATTG-3’

From Qiagen:
QuantiTect Primers
OAS1 (Cat. No. QT00099134)
IFI27 (Cat. No. QT00099274)

Primers used for CHIP
From IDT:
IRF9-F 5´-GACAGTGGCATCCTGATAGTG-3´
IRF9-R 5´-CCCATCCTTGGCTGATCTTAC-3´
c-FOS-F 5´-CCCGACCTCGGGAACAAGGG-3´
c-FOS-R 5´-ATGAGGGGTTTCGGGGATGG-3´
